# Antibiotic Use and the Risk of Hospital-Onset *Clostridioides Difficile* Infection

**DOI:** 10.1001/jamanetworkopen.2025.25252

**Published:** 2025-08-08

**Authors:** Mayan Gilboa, Gili Regev-Yochay, Eyal Meltzer, Ido Cohen, Yovel Peretz, Tal Zilberman-Daniels, Amitai Segev, Sharon Amit, Dafna Yahav, Noam Barda

**Affiliations:** 1 Faculty of Medical and Health Sciences, Tel-Aviv University, Ramat-Aviv, Tel-Aviv, Israel; 2 The Sheba Pandemic Preparedness Research Institute, Sheba Medical Center, Ramat-Gan, Israel; 3 Infection Prevention and Control Unit, Sheba Medical Center, Ramat-Gan, Israel; 4 Internal Medicine C, Sheba Medical Center, Ramat-Gan, Israel; 5 Internal Medicine E, Sheba Medical Center, Ramat-Gan, Israel; 6 Microbiology Laboratory, Sheba Medical Center, Ramat-Gan, Israel; 7 Infectious Diseases Unit, Sheba Medical Center, Ramat-Gan, Israel; 8 Cardiovascular Division, Sheba Medical Center, Tel Hashomer, Ramat-Gan, Israel; 9 ARC Innovation Center, Sheba Medical Center, Tel Hashomer, Ramat Gan, Israel; 10 Software and Information Systems Engineering, Ben-Gurion University of the Negev, Be’er Sheva, Israel; 11 Epidemiology, Biostatistics, and Community Health Sciences, Ben-Gurion University of the Negev, Be’er Sheva, Israel

## Abstract

**Importance:**

*Clostridioides difficile* is a leading cause of health care–associated infections. Understanding the association among *C difficile* carriage, antibiotic use, and infection hazard is essential for infection prevention.

**Objective:**

To evaluate the hazard of *C difficile* infection (CDI) among asymptomatic carriers vs noncarriers of *C difficile* and whether it is associated with antibiotic exposure.

**Design, Setting, and Participants:**

This retrospective cohort study conducted between June 18, 2017, and June 21, 2023, analyzed hospitalizations from Sheba Medical Center in Ramat Gan, Israel, which routinely screens for *C difficile* in high-risk patients admitted to internal medicine. Adult patients (aged >18 years) without active CDI at admission were included.

**Exposure:**

Antibiotic exposure during hospitalization, including specific classes.

**Main Outcomes and Measures:**

The primary outcome was the development of CDI, as confirmed by laboratory testing for *C difficile*. Antibiotic exposure was assessed as a time-varying variable.

**Results:**

The study included 33 756 hospitalizations among 23 001 patients (median [IQR] age, 78 [68-87] years; 52.8% men). *C difficile* infection occurred in 67 of 1624 hospitalizations (4.1%) with positive screening results and in 47 of 32 132 hospitalizations (0.1%) with negative screening results. A positive *C difficile* screening result at admission was associated with a high hazard of infection (hazard ratio [HR], 27.5; 95% CI, 18.7-40.3). Antibiotic exposure was associated with an increased hazard for CDI (HR, 1.98; 95% CI, 1.24-3.16). Piperacillin and tazobactam showed the most pronounced hazard for CDI (HR, 2.18; 95% CI, 1.41-3.36). Among asymptomatic carriers, antibiotic exposure was not significantly associated with a further increase in CDI hazard (HR, 1.07; 95% CI, 0.73-1.58).

**Conclusions and Relevance:**

In this cohort study, carriers of *C difficile* had a substantially higher baseline hazard for hospital-onset CDI. Antibiotic exposure was associated with an increased hazard among noncarriers but was not significantly associated with additional hazard among carriers. These findings suggest that while antibiotic stewardship may reduce CDI risk in noncarriers, additional strategies may be needed for carriers given their elevated baseline risk.

## Key Points

**Question:**

What is the hazard of *Clostridioides difficile* infection (CDI) between asymptomatic carriers compared with noncarriers of *C difficile*, and is this hazard associated with antibiotic exposure?

**Findings:**

In this cohort study of 33 756 hospitalizations among 23 001 patients, asymptomatic carriers had a higher hazard of CDI. Antibiotic exposure, particularly to amoxicillin and clavulanate and piperacillin and tazobactam, was associated with an increased hazard of CDI, especially among noncarriers, with no additional hazard among carriers.

**Meaning:**

These findings suggest that while antibiotic stewardship may be associated with a reduced hazard of CDI in asymptomatic carriers given their already elevated hazard, additional strategies may be warranted.

## Teaser

This cohort study evaluates the risk of hospital-onset *Clostridioides difficile* infection between asymptomatic carriers and noncarriers of *C difficile* upon admission and whether it is associated with antibiotic exposure.

## Introduction

*Clostridioides difficile* is a leading cause of health care–associated infections. Patients with *C difficile* infection (CDI) endure considerable morbidity and mortality, while health care facilities face substantial costs related to treatment and infection control efforts.^1,2,3,4,5^ Despite various preventive strategies, the incidence of CDI remains high, necessitating further exploration of risk factors and targeted interventions.

*C difficile* infection occurs primarily in individuals with disrupted gut microbiota, and antibiotic exposure is a well-recognized risk factor. Broad-spectrum antibiotics, such as fluoroquinolones, clindamycin, and broad-spectrum cephalosporins, substantially increase CDI risk by altering the gut microbiome and creating conditions favorable for *C difficile* colonization and proliferation.^6,7,8^ Moreover, the risk of CDI is influenced by the duration and intensity of antibiotic exposure, with prolonged and multiple courses of antibiotics considered particularly hazardous.^9^ Other risk factors include advanced age, comorbidities, proton pump inhibitor use, and prior CDI events.^10^

In addition to these general risk factors, asymptomatic carriers of *C difficile*, ie, individuals with positive test results for the bacterium without exhibiting symptoms, present unique challenges in health care settings. These carriers not only act as potential reservoirs for transmission but also are at an elevated risk of progressing to clinical CDI.^11,12,13,14,15^ However, the specific interactions among antibiotic use, type, and duration of treatment in this high-risk group remain poorly understood. The aim of our study was to address these knowledge gaps by quantifying the rate of hospital-onset CDI among carriers of *C difficile* compared with noncarriers and by evaluating the role of antibiotic exposure in shaping this risk.

## Methods

### Study Data and Design

This retrospective cohort study used electronic health record (EHR) data of Sheba Medical Center in Ramat Gan, Israel, a large tertiary medical center. The study was approved by the Sheba Medical Center Institutional Review Board, with a waiver of informed consent as the study was deemed a hospital-wide infection prevention and quality improvement initiative that did not pose a risk to patients. The study followed the Strengthening the Reporting of Observational Studies in Epidemiology (STROBE) reporting guideline.^16^

Sheba Medical Center’s EHR data encompass all medical data generated during a hospitalization, including demographic, clinical, and laboratory data. The study period was from June 18, 2017, to June 21, 2023. Since April 2017, we implemented routine *C difficile* screening for high-risk patients admitted to internal medicine departments at Sheba Medical Center. Eligible patients were adults (aged >18 years); hospitalized in internal medicine wards; and deemed high risk for *C difficile* carriage, ie, hospitalized within the past 6 months or transferred from another hospital or a long-term-care facility. Detailed information on the identification process and EHR-based flagging criteria is available in eMethods 1 in Supplement 1.

Patients who screened positive for *C difficile* were placed in contact isolation, and an EHR note was added to indicate their high risk for CDI. We excluded patients who had active CDI at the time of admission or within 48 hours (as this was deemed unrelated to exposures that occurred during the current hospitalization), as well as patients who were transferred to the intensive care unit (ICU) before the onset of CDI. All patients were followed up from 2 days after hospitalization (time 0) until death, discharge, day 21 of the hospitalization, admission to the ICU, or CDI, whichever was earliest. Patients who were transferred to the ICU before the onset of CDI were included in the cohort, but follow-up was censored at the time of ICU transfer (ie, only data from before ICU admission were included). If a patient had multiple readmissions in the study period, these admissions were included in the analysis. We included only the first 21 days from each hospitalization, as longer hospitalizations at Sheba Medical Center typically represent patients with nonacute conditions awaiting institutional placement and with limited antibiotic exposure, which could bias hazard estimates.

All study variables were extracted from the EHR with the assistance of a professional data analyst (Y.P.). A clinician (M.G.) manually validated a random 5% sample of records against the original EHRs to confirm data accuracy.

### Study Variables

The exposure of interest was treatment with antibiotics during the hospitalization. Antibiotic use was modeled as a time-varying exposure, in which patients would contribute person-time to the group that did not receive antibiotics prior to exposure and subsequently would contribute person-time to the respective antibiotic group after exposure. In all cases, we treated antibiotic exposure as accumulating throughout the hospitalization, such that exposure on a given day always considered antibiotics given from the start of the hospitalization until that day. Any administration of an antibiotic on a given calendar day was counted as 1 exposed day, regardless of dose frequency or amount. First, we modeled any exposure to antibiotics during the hospitalization as a binary variable. Next, we modeled the cumulative number of days exposed to any antibiotic.

We then considered antibiotics in separate classes: penicillins, cephalosporins (divided into first and second generation vs third and fourth generation), amoxicillin and clavulanate, piperacillin and tazobactam, carbapenem, aminoglycosides, quinolones, clindamycin, intravenous vancomycin, and other antibiotics (including tetracyclines, trimethoprim-sulfamethoxazole, macrolides, and chloramphenicol). Exposure to the different classes was again modeled first as a binary variable (ie, any exposure to this antibiotic class during hospitalization until the current day) and as a summation of days of exposure (ie, number of days exposed to this antibiotic class during hospitalization until the current day). Finally, we modeled the number of different antibiotic classes exposed to during the hospitalization. A more detailed explanation is included in eMethods 2 in Supplement 1.

The outcome of interest was CDI, defined as a laboratory report of an unformed stool with a positive polymerase chain reaction (PCR) result (Xpert *C difficile*/Epi; Cepheid), together with a positive enzyme immunoassay (EIA) result for either glutamate dehydrogenase or *C difficile* toxin, which were performed on all samples. *C difficile* infection testing was clinically driven. For all positive results, an infection prevention nurse reviewed the EHR to confirm the presence of 3 or more unformed stools in 24 hours and no laxative use per Centers for Disease Control and Prevention criteria.^17^

Covariates for adjustment were selected based on domain expertise, and included age, sex, Charlson Comorbidity Index score,^18^ functional state as reported by the nursing staff (binary variable of independent in or requires assistance with activities of daily living), immunosuppression (binary variable, with patients classified as immunosuppressed if they had a diagnosis of a solid tumor, leukemia, lymphoma, AIDS, hematopoietic or solid organ transplant, or chronic corticosteroid treatment equivalent to >20 mg/d of prednisone), and use of protein pump inhibitors. All eligibility criteria and covariates for adjustment were extracted from the EHR before the start of follow-up.

We also adjusted for admission *C difficile* screening results. Screening for *C difficile* was based on a rectal swab (Stuarts Swab; Copan Diagnostics), with a PCR assay used to detect sequences of the genes for toxin B (*tcdB*) and binary toxin (*cdt*) (Xpert *C difficile*/Epi). This assay has been validated and approved for testing unformed stool for CDI diagnosis; its use for screening, though, is done off label. Patients were screened for *C difficile* within 24 hours of admission. Performance of the screening was not related to receipt of antibiotics during the index hospitalization. Additionally, we performed a subgroup analysis for the patients with positive admission *C difficile* rectal swab screening results. An additional sensitivity analysis was done by repeating analyses using a modified outcome definition that excluded EIA testing for glutamate dehydrogenase, in which patients were considered to have CDI only if they had both a positive stool PCR result and a *C difficile* toxin–positive EIA result.

### Statistical Analysis

The study population was described for relevant variables at the level of each distinct hospitalization, using appropriate statistics for each variable. The description was additionally stratified for *C difficile* screening results at admission.

To analyze the study data, we opted for time-to-event (survival) analysis. To this end, data were arranged in a counting process (Anderson-Gill) format. Follow-up began at day 3 of each hospitalization and lasted until the outcome or censoring due to death, discharge, or admission to the ICU.

*C difficile* screening results at admission were modeled separately as an exposure and used for stratification of the analysis of the antibiotic exposures. With an aim to explore a nonlinear association between the cumulative exposure and the hazard of CDI, we further modeled days of treatment, a continuous variable, as a smoothing restricted cubic spline.

The model used throughout was a multivariable Cox proportional hazards model. From each model, we reported as the outcome of interest the exponentiated coefficient of the exposure, interpreted as the hazard ratio (HR). Because patients were allowed to contribute multiple hospitalizations, data were analyzed as hierarchical, clustered at the level of the individual, using robust standard errors. We additionally drew survival curves based on the adjusted models, using representative baseline covariate values (the median values from the sample) and simple exposure histories starting at day 3 and lasting until day 21.

Two sensitivity analyses were performed. First, we repeated the analysis with a more specific definition for the outcome (CDI positivity defined by a positive toxin test result). Second, we used only the first hospitalization of each individual during the study period.

Because we considered the purpose of this study etiologic (as opposed to prognostic),^19,20^ we performed the primary analysis without accounting for competing risks. In the context of this study, missing data were only possible for the variable Charlson Comorbidity Index score, as it is known to be rare in Sheba Medical Center’s database (<1%) and assumed to be missing completely at random. Thus, we performed complete case analysis. More specific details regarding the structure of the dataset are included in eMethods 2 in Supplement 1.

The analyses were performed using the survival package, version 3.5-8 in R, version 4.4.0 (R Foundation for Statistical Computing). A link to the analysis code for the main analysis is available in eMethods 2 in Supplement 1. Results were considered significant if the 95% CI did not include 1.

## Results

**Figure 1.**
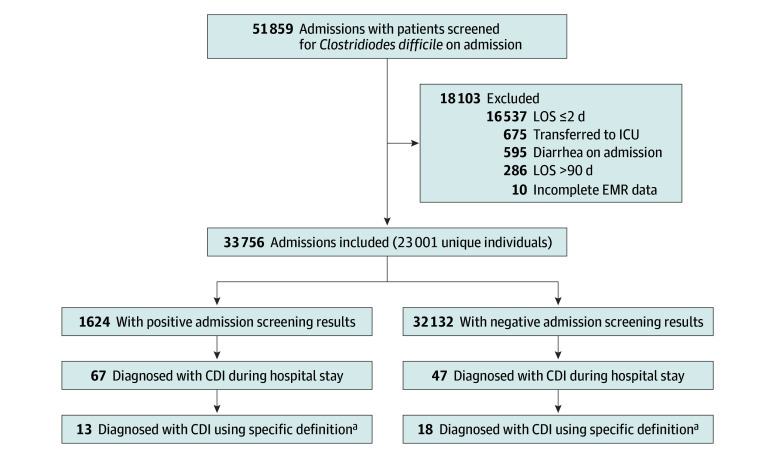
Study Population Flowchart CDI indicates *Clostridioides difficile *infection; ICU, intensive care unit; LOS, length of stay. ^a^Specific CDI definition is positive polymerase chain reaction and enzyme immunoassay results for *C difficile* toxin.

Overall, the study included 23 001 distinct individuals (median [IQR] age, 78 [68-87] years; 47.5% female and 52.5% male) contributing 33 756 hospitalizations, totaling 201 631 hospital days (Figure 1). The median Charlson Comorbidity Index score was 6 (IQR, 4-8), and 58.7% were considered not independent in their baseline functional capacity. A total of 1624 admissions (4.8%) had a positive screening result for *C difficile *(hereafter referred to as carriers). These patients were more likely to have a nonindependent functional state (74.6% vs 57.8% with a negative screening result) (Table 1). During the study period, 15 396 patients met the criteria for screening. Of these patients, 11 334 (73.6%) were screened by rectal swab. Among the collected swabs, 91.5% yielded valid results, while 8.5% were disqualified due to insufficient fecal material.

**Table 1.** Study Population^a^

Variable	All hospitalizations (N = 33 756)	*Clostridioides difficile* screening result	
		Negative (n = 32 132)	Positive (n = 1624)
Age, median (IQR), y	78 (68-87)	78 (67-87)	79 (68-87)
Sex, No. (%)			
Female	16 044 (47.5)	15 278 (47.5)	766 (47.2)
Male	17 712 (52.5)	16 854 (52.5)	858 (52.8)
Charlson Comorbidity Index score, median (IQR)^b^	6 (4-8)	6.0 (4.0-8.0)	6.0 (4.0-9.0)
Nonindependent functional state, No. (%)	19 798 (58.7)	18 587 (57.8)	1211 (74.6)
Immunosuppression, No. (%)^c^	523 (1.5)	493 (1.5)	30 (1.8)
Use of proton pump inhibitors, No. (%)	15 271 (45.2)	14 497 (45.1)	774 (47.7)
Any antibiotic use, No. (%)	22 499 (66.7)	21 264 (66.2)	1235 (76.0)
No. of antibiotics received, No. (%)			
0	11 257 (33.3)	10 868 (33.8)	389 (24.0)
1	7518 (22.3)	7099 (22.1)	419 (25.8)
2	8069 (23.9)	7655 (23.8)	414 (25.5)
3	4654 (13.8)	4387 (13.6)	267 (16.4)
4	1611 (4.8)	1521 (4.7)	90 (5.5)
5	505 (1.5)	470 (1.5)	35 (2.2)
6	121 (0.4)	111 (0.3)	10 (0.6)
7	17 (<0.1)	17 (<0.1)	0
8	4 (<0.1)	4 (<0.1)	0
Antibiotics received, No. (%)			
Aminoglycosides	913 (2.7)	863 (2.7)	50 (3.1)
Amoxicillin and clavulanate	5699 (16.9)	5347 (16.6)	352 (21.7)
First- and second-generation cephalosporins	2219 (6.6)	2121 (6.6)	98 (6.0)
Third- and fourth-generation cephalosporins	12 852 (38.1)	12 175 (37.9)	677 (41.7)
Carbapenems	2294 (6.8)	2141 (6.7)	153 (9.4)
Clindamycin	859 (2.5)	821 (2.6)	38 (2.3)
Penicillins	1806 (5.4)	1718 (5.3)	88 (5.4)
Piperacillin and tazobactam	5785 (17.1)	5401 (16.8)	384 (23.6)
Quinolones	8168 (24.2)	7761 (24.2)	407 (25.1)
Intravenous vancomycin	2846 (8.4)	2645 (8.2)	201 (12.4)
Other antibiotics^d^	3953 (11.7)	3763 (11.7%)	190 (11.7)

^a^ Each row represents a hospitalization (patients may have multiple entries).

^b^ Charlson Comorbidity Index scores range from 0 to 37, with higher scores indicating a greater burden of disease.

^c^ Immunosuppression includes malignant tumor, transplant, AIDS, or chronic corticosteroid use (equivalent to >20 mg/d prednisone).

^d^ Other antibiotics include tetracyclines, trimethoprim and sulfamethoxazole, macrolides, and chloramphenicol.

Overall, 67 of 1624 carriers (4.1%) and 47 of 32 132 noncarriers (ie, admissions with negative screening results) (0.1%) developed CDI (Figure 1). To evaluate potential bias in testing behavior, we assessed the frequency and outcomes of CDI testing by colonization status. Among the 1624 carriers, 251 CDI tests were sent (15.5%) compared with 2067 tests among the 32 132 noncarriers (6.4%). Despite more frequent testing in carriers, the positivity rate was markedly higher at 26.7% (67 of 251) in carriers vs 2.3% (46 of 2067) in noncarriers.

In the entire cohort, exposure to any antibiotic was associated with an increased hazard for CDI (HR, 1.98; 95% CI, 1.24-3.16), with each additional day of exposure to antibiotics having an HR of 1.08 (95% CI, 1.03-1.13). Exposure to each additional antibiotic group increased the hazard (HR, 1.21; 95% CI, 1.08-1.37) (Table 2; eTable 1 in Supplement 1). The median hospital lengths of stay were 4 days (IQR, 3-7 days) in which antibiotics were not administered and 6 days (IQR, 4-13 days) in which antibiotics were administered. The distribution of the number of exposure days per antibiotic class is shown in eFigure 1 in Supplement 1.

**Table 2.** Association Between Exposure to Any Antibiotic and CDI

Exposure	HR (95% CI)	
	Crude	Adjusted^a^
**Original CDI definition** ^b^		
Entire population		
Any antibiotic treatment (binary)	2.06 (1.28-3.32)	1.98 (1.24-3.16)
No. of treatment days (continuous)	1.08 (1.03-1.13)	1.08 (1.03-1.13)
No. of antibiotic drugs (continuous)	1.24 (1.10-1.40)	1.21 (1.08-1.37)
Positive *Clostridiodes difficile* screen		
Any antibiotic treatment (binary)	1.04 (0.58-1.86)	1.07 (0.61-1.87)
No. of treatment days (continuous)	1.03 (0.96-1.11)	1.04 (0.97-1.11)
No. of antibiotic drugs (continuous)	1.01 (0.84-1.22)	1.03 (0.86-1.24)
**Toxin EIA-positive result only**		
Entire population		
Any antibiotic treatment (binary)	2.09 (0.87-5.01)	1.85 (0.81-4.19)
No. of treatment days (continuous)	1.11 (1.03-1.21)	1.10 (1.02-1.18)
No. of antibiotic drugs (continuous)	1.12 (0.93-1.34)	1.07 (0.90-1.26)
Positive *C difficile* screen		
Any antibiotic treatment (binary)	1.07 (0.39-2.92)	1.12 (0.43-2.96)
No. of treatment days (continuous)	1.03 (0.91-1.16)	1.05 (0.93-1.18)
No. of antibiotic drugs (continuous)	0.94 (0.68-1.30)	0.98 (0.71-1.35)

Abbreviations: CDI, *Clostridioides difficile* infection; EIA, enzyme immunoassay; HR, hazard ratio.

^a^ Hazard ratios are from Cox models adjusted for age, sex, Charlson Comorbidity Index score, functional status, immunosuppression, and protein pump inhibitor use.

^b^ Defined per Centers for Disease Control and Prevention testing criteria (≥3 unformed stools in 24 hours, no laxatives). Original CDI definition is unformed stool with positive polymerase chain reaction and EIA results for glutamate dehydrogenase or *C difficile* toxin.

Considering antibiotics separately, we found that exposure to amoxicillin and clavulanate was associated with an increased hazard of CDI (HR, 1.72; 95% CI, 1.12-2.64), with each additional day of exposure having an HR of 1.15 (95% CI, 1.08-1.23). Exposure to piperacillin and tazobactam also was associated with an increased hazard (HR, 2.18; 95% CI, 1.41-3.36), with each additional day having an HR of 1.13 (95% CI, 1.07-1.20). Other antibiotics were not significantly associated with an increased hazard (Table 3; eTables 2 and 3 in Supplement 1). Survival curves are included in eFigure 2 in Supplement 1 to help visualize the association of various antibiotic exposures.

**Table 3.** Association Between Exposure to Specific Antibiotic Classes and CDI

Antibiotic exposure	HR (95% CI)^a^							
	Original CDI definition^b^^,^^c^				Specific CDI definition^b^^,^^d^			
	Entire population		Positive *Clostridiodes difficile* screen		Entire population		Positive *C difficile* screen	
	Any exposure	No. of days	Any exposure	No. of days	Any exposure	No. of days	Any exposure	No. of days
Aminoglycosides	1.54 (0.68-3.49)	0.98 (0.82-1.17)	0.97 (0.21-4.55)	0.75 (0.45-1.23)	1.02 (0.12-8.59)	0.75 (0.43-1.36)	ND	ND
Amoxicillin and clavulanate	1.72 (1.12-2.64)	1.15 (1.08-1.23)	1.1 (0.62-1.95)	1.13 (1.04-1.23)	2.15 (1.05-4.43)	1.16 (1.04-1.30)	1.15 (1.01-1.30)	ND
First- and second-generation cephalosporins	1.00 (0.45-2.23)	1 (0.86-1.17)	0.76 (0.23-2.54)	0.99 (0.75-1.32)	0.41 (0.05-3.06)	0.84 (0.52-1.36)	ND	ND
Third- and fourth-generation cephalosporins	1.13 (0.75-1.69)	1.01 (0.93-1.09)	0.76 (0.45-1.31)	0.91 (0.79-1.04)	1.06 (0.48-2.34)	1.03 (0.90-1.18)	0.95 (0.76-1.18)	ND
Carbapenem	1.27 (0.72-2.26)	1.03 (0.92-1.14)	1.54 (0.76-3.14)	1.12 (0.97-1.28)	0.88 (0.26-2.93)	0.93 (0.73-1.18)	1.10 (0.89-1.37)	1.07 (0.80-1.43)
Clindamycin	0.56 (0.14-2.32)	0.79 (0.51-1.25)	0.68 (0.10-4.69)	0.66 (0.36-1.23)	0.88 (0.13-6.09)	0.75 (0.42-1.31)	0.87 (0.64-1.17)	0.84 (0.34-2.04)
Piperacillin and tazobactam	2.18 (1.41-3.36)	1.13 (1.07-1.20)	1.70 (0.95-3.01)	1.11 (1.01-1.21)	1.40 (0.58-3.38)	1.08 (0.95-1.22)	1.24 (0.51-2.99)	1.09 (0.91-1.29)
Other penicillins	0.52 (0.18-1.45)	0.95 (0.77-1.18)	0.31 (0.04-2.16)	0.46 (0.16-1.29)	0.42 (0.06-3.25)	1.02 (0.75-1.37)	ND	ND
Quinolones	0.88 (0.55-1.41)	0.98 (0.91-1.07)	0.95 (0.50-1.83)	1.01 (0.89-1.13)	1.08 (0.45-2.58)	1.02 (0.89-1.17)	1.38 (0.48-4.01)	1.05 (0.89-1.24)
Intravenous vancomycin	1.20 (0.69-2.09)	1.05 (0.94-1.17)	0.86 (0.92-1.27)	1.08 (0.91-1.28)	1.26 (0.4-3.94)	1.11 (0.92-1.33)	0.99 (0.34-2.9)	1.12 (0.92-1.35)
Other antibiotics^e^	0.88 (0.48-1.62)	0.99 (0.86-1.15)	0.99 (0.80-1.21)	0.99 (0.80-1.22)	0.47 (0.11-1.97)	1.01 (0.73-1.39)	ND	ND

Abbreviations: CDI, *Clostridioides difficile* infection; HR, hazard ratio; ND, insufficient data to estimate association.

^a^ Hazard ratios are from Cox models adjusted for age, sex, Charlson Comorbidity Index score, functional status, immunosuppression, and protein pump inhibitor use.

^b^ Testing for CDI followed Centers for Disease Control and Prevention guidelines (≥3 unformed stools in 24 hours, no laxatives).

^c^ Original CDI definition is unformed stool with positive polymerase chain reaction and enzyme immunoassay results for glutamate dehydrogenase or *C difficile* toxin.

^d^ Specific CDI definition is positive polymerase chain reaction and enzyme immunoassay results for *C difficile* toxin.

^e^ Other antibiotics include tetracyclines, trimethoprim and sulfamethoxazole, macrolides, and chloramphenicol.

**Figure 2.**
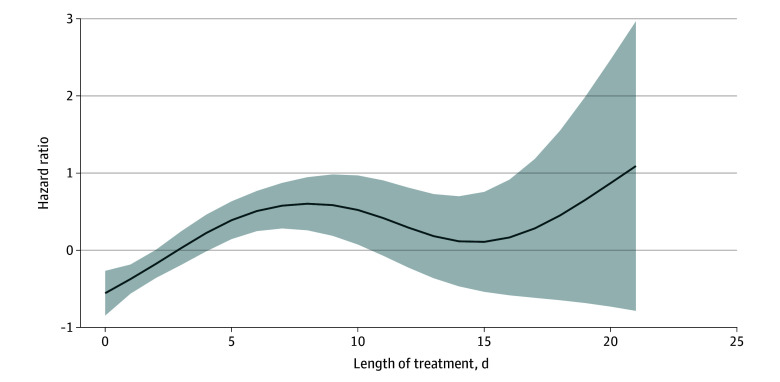
Flexible Modeling of the Association Between Days of Treatment With Any Antibiotic and *Clostridioides Difficile* Infection (CDI) Hazard ratios were estimated using Cox models adjusted for age, sex, Charlson Comorbidity Index score, functional status, immunosuppression, and protein pump inhibitor use. Associations were modeled using restricted cubic splines. Shading indicates the 95% CI.

By modeling days of treatment flexibly using a cubic spline, we found that the hazard did not increase monotonically. Rather, it increased over the first few days of receiving antibiotics, then remained constant and finally increased again for long (>14 days) treatment durations (Figure 2).

A positive *C difficile* screening result at admission was found to be associated with CDI, showing a large HR during hospitalization of 27.5 (95% CI, 18.7-40.3). By restricting the analyses to the 1624 carriers, we found no association between CDI and exposure to antibiotics (overall and in separate groups) in this population (Table 2; Table 3).

After repeating the analysis with a more specific definition for the outcome (CDI positivity defined by a positive toxin test result), we found that a positive screening result was associated with a large hazard for this outcome (HR, 32.6; 95% CI, 16.0-66.5). Repeating the analysis for antibiotic exposure with this more specific outcome yielded qualitatively similar results, though with less statistical power (Table 2; Table 3).

We also performed a quantitative bias analysis assuming 80% assay sensitivity to account for potential misclassification of patients with *C difficile* colonization. The adjusted HRs remained consistent across all 10 iterations (eTable 4 in Supplement 1). After repeating the analysis using only the first hospitalization of each individual, we found that the results remained similar (eTables 5 and 6 in Supplement 1).

## Discussion

This retrospective cohort study highlights the complex association among antibiotic exposure, asymptomatic *C difficile* carriage, and hospital-onset CDI. In a large cohort of hospitalized patients, we found that while antibiotic use was significantly associated with increased CDI hazard in noncarriers, the association was less pronounced among asymptomatic carriers, possibly given their inherently high baseline hazard.

Our analysis confirms that antibiotic exposure is a major risk factor for CDI, consistent with previous research. β-Lactam/β-lactamase inhibitor combinations (BLBLIs), particularly piperacillin and tazobactam, were associated with the highest hazards, with each additional day of exposure compounding this association. This finding aligns with that of Webb et al^9^ and Schechner et al,^21^ who identified specific antibiotic classes and prolonged use as significant contributors to CDI hazard. In these studies, BLBLIs were found to be among the highest risk antibiotics for CDI, along with carbapenems and broad-spectrum cephalosporins. Schechner et al reported an increase of more than 2% per day of BLBLI use, similar only to carbapenems and cephalosporins. Webb et al reported an odds ratio of 5.1 (95% CI, 4.4-6.0) for CDI within 60 days of piperacillin and tazobactam administration in an adjusted analysis. In this study, the odds of CDI were found to be associated with a 13% increase per day of antibiotic therapy. Our study supports these findings by showing a nonlinear association between antibiotic duration and CDI hazard, suggesting that the association may stabilize after initial exposure but rise again with extended treatment (>14 days).

Our analysis did not find significant associations between CDI and certain antibiotic classes traditionally considered high risk, such as third- and fourth-generation cephalosporins, carbapenems, fluoroquinolones, and clindamycin. This observation is consistent with recent studies suggesting a more pronounced association between CDI and BLBLIs, while the association with classical agents may be diminished in some settings.^22,23,24^ Other studies, however, reported associations with both BLBLIs and traditional agents.^25^ The absence of an observed association in our cohort may reflect local prescribing patterns, antibiotic stewardship efforts, or limited exposure to these agents during hospitalization.

Considering the growing literature supporting shorter antibiotic courses for various infections,^26,27^ along with the association between antibiotic use and CDI that appears more pronounced after day 14, duration of therapy should be a focus of antibiotic stewardship programs aimed at reducing CDI rates. Such a focus is particularly important for patients who are not carriers of *C difficile* upon admission (>95% of all admissions).

Among asymptomatic carriers, we found a 27.5-fold higher hazard for CDI, underscoring the substantial baseline vulnerability of this group. Interestingly, we observed no significant additional hazard from antibiotic exposure during hospitalization in this group, contrasting with findings from Poirier et al,^28^ who identified antibiotic use as a predictor of CDI among carriers. This discrepancy may be explained by differences in study design. The study by Poirier et al included antibiotic exposure during hospitalization and in the 3 months prior to admission, potentially capturing a wider view of modifiable risk. In our study, carriers were already at such an elevated baseline risk that changes in antibiotic exposure during hospitalization may have had a minimal influence on CDI risk. This explanation aligns with prior findings that up to 40% of asymptomatic carriers develop CDI during hospitalization,^13^ suggesting that these patients may already be on a trajectory toward infection, with antibiotic use during hospitalization contributing little additional risk. In addition, it supports the hypothesis that late-stage *C difficile* carriage may represent a predisease state rather than benign colonization. Studies have shown that carriers already exhibit altered gut microbiota characterized by reduced species richness and diversity.^29^ These findings underscore the importance of the timeline of antibiotic exposure prior to hospitalization, which may play a pivotal role in carriage acquisition and might be the key driver of the increased CDI hazard observed in this population.

Although knowledge of colonization status may have influenced testing, the positivity rate was substantially higher among carriers (27%) compared with noncarriers (2.3%), despite only modestly higher testing rates (15% vs 6.5%). This finding suggests that increased testing may reflect true elevated risk rather than lower testing thresholds.

Among carriers, preventive measures in addition to antibiotic stewardship should be evaluated in hospitalized patients and may include probiotics, monoclonal antibodies, or biotherapies.^30,31^ However, further research is necessary to evaluate the potential effectiveness of these interventions, their association with microbiome alterations, and potential changes in antimicrobial resistance patterns among patients and within the hospital environment.

### Limitations

Several limitations in this study warrant consideration. Residual confounding may persist despite adjustments for patient demographics, comorbidities, and functional status; that is, it is possible that an unadjusted correlation still exists between choice of antibiotic and a patient’s overall condition. Additionally, the lack of community-based data limited our ability to account for prehospital antibiotic exposure, which may influence both colonization and infection risks. Including such earlier exposure timelines could provide a more comprehensive understanding of CDI progression. We also did not evaluate interactions between antibiotic classes, as the number of combinations and the limited sample size precluded reliable analysis.

A further limitation of this study was its sampling scope. The analysis included only high-risk patients admitted to internal medicine wards at a single academic medical center. This population was predominantly older adults, medically complex, and functionally dependent, which may limit generalizability and may explain differences from other cohorts. Screening criteria were based on prior work identifying risk factors for *C difficile* carriage.^14^

Finally, the PCR assay used, although US Food and Drug Administration approved for the diagnosis of symptomatic infection, has not been validated for detecting asymptomatic *C difficile* colonization. This off-label use may have resulted in some misclassification of carrier status. To account for this potential limitation, we performed a quantitative bias analysis assuming an 80% sensitivity of the assay. The results showed that our primary findings remained consistent across varying assumptions (eTable 4 in Supplement 1).

## Conclusions

This cohort study emphasizes the need for nuanced CDI prevention strategies that address distinct risks in carriers and noncarriers of *C difficile*. While antibiotic stewardship is crucial for reducing CDI in general, additional approaches are needed for carriers to mitigate their substantially high baseline risk. Further research should explore modifiable factors beyond antibiotic use to improve outcomes in patients with CDI and to reduce the overall burden of CDI in health care settings. Shortened duration of antibiotic therapy and restriction of BLBLIs may reduce CDI among hospitalized patients.

## Notes

Supplement 1. **eMethods 1.** Identification of High-Risk Patients and Screening Process **eMethods 2.** Time-Varying Antibiotic Exposure Modeling **eTable 1.** Association Between Exposure to Any Antibiotic and *Clostridioides Difficile* Infection (CDI), for Patients With a Negative Screening Result, Adjusted **eTable 2.** Association Between Exposure to Specific Antibiotic Classes and *Clostridioides Difficile* Infection (CDI), Crude **eTable 3.** Association Between Exposure to Specific Antibiotic Classes and *Clostridiodes Difficile* Infection (CDI) for Those With Negative Screening Results **eTable 4.** Sensitivity Analysis Accounting for 80% Assay Sensitivity: Adjusted Hazard Ratios for CDI by Antibiotic Exposure Across 10 Iterations **eTable 5.** Association Between Exposure to Any Antibiotic and *Clostridioides Difficile* Infection (CDI), Only the First Hospitalization for Each Patient Included **eTable 6.** Association Between Exposure to Specific Antibiotic Classes and *Clostridioides Difficile* Infection (CDI), Only the First Hospitalization for Each Patient Included **eFigure 1.** Antibiotic Exposure Histograms **eFigure 2.** Survival Curves

Supplement 2.
Data Sharing Statement
