# Supplementary material for: Antibiotic Use and the Risk of Hospital-Onset Clostridioides Difficile Infection
Source: JAMA Netw Open. 2025 Aug 8;8(8):e2525252. doi: 10.1001/jamanetworkopen.2025.25252 (PMC12334957; doi:10.1001/jamanetworkopen.2025.25252)
Supplement: Supplement 1. — eMethods 1. Identification of High-Risk Patients and Screening Process eMethods 2. Time-Varying Antibiotic Exposure Modeling eTable 1. Association Between Exposure to Any Antibiotic and Clostridioides Difficile Infection (CDI), for Patients With a Negative Screening Result, Adjusted eTable 2. Association Between Exposure to Specific Antibiotic Classes and Clostridioides Difficile Infection (CDI), Crude eTable 3. Association Between Exposure to Specific Antibiotic Classes and Clostridiodes Difficile Infection (CDI) for Those With Negative Screening Results eTable 4. Sensitivity Analysis Accounting for 80% Assay Sensitivity: Adjusted Hazard Ratios for CDI by Antibiotic Exposure Across 10 Iterations eTable 5. Association Between Exposure to Any Antibiotic and Clostridioides Difficile Infection (CDI), Only the First Hospitalization for Each Patient Included eTable 6. Association Between Exposure to Specific Antibiotic Classes and Clostridioides Difficile Infection (CDI), Only the First Hospitalization for Each Patient Included eFigure 1. Antibiotic Exposure Histograms eFigure 2. Survival Curves [file jamanetwopen-e2525252-s001.pdf]

Supplemental Online Content

Gilboa M, Regev-Yochay G, Meltzer E, et al. Antibiotic use and the risk of hospital-onset *Clostridioides difficile* infection. *JAMA Netw Open*. 2025;8(8):e2525252.
doi:10.1001/jamanetworkopen.2025.25252

eMethods 1. Identification of High-Risk Patients and Screening Process

eMethods 2. Time-Varying Antibiotic Exposure Modeling

eTable 1. Association Between Exposure to Any Antibiotic and *Clostridioides Difficile* Infection (CDI), for Patients With a Negative Screening Result, Adjusted

eTable 2. Association Between Exposure to Specific Antibiotic Classes and *Clostridioides Difficile* Infection (CDI), Crude

eTable 3. Association Between Exposure to Specific Antibiotic Classes and *Clostridiodes Difficile* Infection (CDI) for Those With Negative Screening Results

eTable 4. Sensitivity Analysis Accounting for 80% Assay Sensitivity: Adjusted Hazard Ratios for CDI by Antibiotic Exposure Across 10 Iterations

eTable 5. Association Between Exposure to Any Antibiotic and *Clostridioides Difficile* Infection (CDI), Only the First Hospitalization for Each Patient Included

eTable 6. Association Between Exposure to Specific Antibiotic Classes and *Clostridioides Difficile* Infection (CDI), Only the First Hospitalization for Each Patient Included

eFigure 1. Antibiotic Exposure Histograms

eFigure 2. Survival Curves

This supplemental material has been provided by the authors to give readers additional information about their work.

© 2025 Gilboa M et al. *JAMA Network Open*.

eMethods 1. Identification of High-Risk Patients and Screening Process

High-risk patients were automatically identified by the hospital's electronic medical record (EMR) system based on predefined criteria: (1) hospitalization within the previous 6 months, (2) transfer from another acute care hospital, or (3) admission from a long-term care facility. When a patient met any of these criteria, the EMR system automatically flagged them and issued a *C. difficile* screening order at admission.

© 2025 Gilboa M et al. *JAMA Network Open*.

eMethods 2. Time-Varying Antibiotic Exposure Modeling

To facilitate the analysis, the dataset was arranged in a "long" (Anderson-Gill) format, with each row pertaining to a single day from a single hospitalization. Covariates were constant throughout a patient's hospitalization, while antibiotic exposure by antibiotic family was treated as a time-varying exposure, changing from day to day, increasing by 1 for any day in which this antibiotic was given to the patient.

For example, if a patient was hospitalized for five days and treated with a Quinolone in the fourth and fifth day, they would have three rows in the data (as the first two days of hospitalization are discarded), with exposure to Quinolones marked 0 for the third day, 1 for the fourth day, and 2 for the fifth day.

This "cumulative" data at the level of a single antibiotic family was used directly as an exposure, and also used to generate the other computed exposures described in the Methods section:

1. "Binary" exposure at the level a single antibiotic family was defined as any day in which that family has a value equal to or greater than 1.
2. Exposure to "any antibiotic" was defined as a day in which any antibiotic family had a value equal to or greater than 1.
3. Cumulative number of antibiotic families exposed to was defined as the number of antibiotic families with a value equal to or greater than 1.
4. Days of treatment was defined as the number of days in which a patient received at least a single antibiotic family.

As described in the Methods section, we also directly model the screen results. Additionally, analyses were repeated for the sensitivity analyses using the more specific outcome, stratified by screening results, only for the first hospitalization, and in a quantitative bias analysis addressing the imperfect sensitivity of the screening process.

The main analysis code is available at:

https://github.com/noambard/CDI_antibiotics

eTable 1. Association Between Exposure to Any Antibiotic and *Clostridioides Difficile* Infection (CDI), for Patients With a **Negative Screening** Result, Adjusted

Exposure	Hazard Ratio (95% CI)
**Crude**	
**Original CDI Definition**	

© 2025 Gilboa M et al. *JAMA Network Open*.

Exposure	Hazard Ratio (95% CI)
Any antibiotic treatment (Binary)	3.14 (1.24-7.92)
Number of treatment days (Continuous)	1.08 (1.02-1.16)
Number of antibiotic drugs (Continuous)	1.36 (1.14-1.62)
**Toxin EIA positive only**	
Any antibiotic treatment (Binary)	3.88 (0.52-29.01)
Number of treatment days (Continuous)	1.16 (1.04-1.29)
Number of antibiotic drugs (Continuous)	1.17 (0.98-1.41)
**Adjusted**	
**Original CDI Definition**	
Any antibiotic treatment (Binary)	3.22 (1.26-8.23)
Number of treatment days (Continuous)	1.09 (1.02-1.16)
Number of antibiotic drugs (Continuous)	1.34 (1.12-1.6)
**Toxin EIA positive only**	
Any antibiotic treatment (Binary)	3.47 (0.54-22.37)
Number of treatment days (Continuous)	1.05 (0.93-1.18)
Number of antibiotic drugs (Continuous)	1.14 (1.04-1.26)

CI – confidence interval

Hazard ratios from Cox models adjusted for age, sex, Charlson score, functional status, immunosuppression, and PPI use. CDI was defined per CDC testing criteria. See Methods for full diagnostic definitions

© 2025 Gilboa M et al. *JAMA Network Open*.

eTable 2. Association Between Exposure to Specific Antibiotic Classes and *Clostridioides Difficile* Infection (CDI), Crude

	**Entire Population**		**Positive CD Screen**		**Negative CD Screen**	
Exposure	Any Exposure	Number of Days	Any Exposure	Number of Days	Any Exposure	Number of Days
Aminoglycosides	1.59 (0.7-3.58)	0.99 (0.83-1.18)	0.96 (0.21-4.43)	0.74 (0.46-1.2)	2.31 (0.89-6.03)	1.04 (0.88-1.24)
Amoxicillin/Clavulanate	1.8 (1.16-2.77)	1.16 (1.08-1.24)	1.14 (0.64-2.05)	1.13 (1.04-1.23)	2.13 (1.11-4.1)	1.13 (1.01-1.26)
First and second generation Cephalosporins	0.96 (0.43-2.14)	1 (0.86-1.16)	0.78 (0.23-2.59)	0.99 (0.74-1.32)	1.19 (0.41-3.47)	1 (0.85-1.18)
Third and fourth generation Cephalosporins	1.13 (0.77-1.67)	1.01 (0.93-1.09)	0.79 (0.47-1.33)	0.91 (0.79-1.04)	1.47 (0.84-2.59)	1.05 (0.96-1.16)
Carbapenem	1.35 (0.76-2.41)	1.04 (0.93-1.15)	1.48 (0.74-2.98)	1.1 (0.96-1.26)	1.01 (0.4-2.55)	0.95 (0.81-1.1)
Clindamycin	0.57 (0.14-2.35)	0.8 (0.5-1.26)	0.72 (0.1-5.12)	0.68 (0.37-1.24)	0.61 (0.08-4.77)	0.9 (0.61-1.35)
Other Penicillin	0.54 (0.2-1.5)	0.96 (0.78-1.18)	0.29 (0.04-1.97)	0.45 (0.16-1.26)	0.84 (0.25-2.81)	1.03 (0.88-1.2)
Piperacillin/Tazobactam	2.24 (1.46-3.42)	1.14 (1.07-1.21)	1.59 (0.91-2.75)	1.09 (0.99-1.2)	2.43 (1.28-4.62)	1.14 (1.05-1.23)
Quinolones	0.85 (0.53-1.36)	0.98 (0.9-1.07)	0.89 (0.47-1.7)	1 (0.89-1.13)	0.79 (0.38-1.62)	0.98 (0.86-1.1)
Vancomycin IV	1.26 (0.73-2.15)	1.06 (0.96-1.18)	0.84 (0.41-1.71)	1.07 (0.91-1.26)	1.46 (0.67-3.18)	1.01 (0.89-1.15)

© 2025 Gilboa M et al. *JAMA Network Open*.

	Entire Population		Positive CD Screen		Negative CD Screen	
Exposure	Any Exposure	Number of Days	Any Exposure	Number of Days	Any Exposure	Number of Days
Other antibiotics	0.92 (0.51-1.68)	1 (0.87-1.16)	1.04 (0.47-2.31)	0.99 (0.8-1.23)	0.84 (0.33-2.11)	1.01 (0.84-1.21)

© 2025 Gilboa M et al. *JAMA Network Open*.

eTable 3. Association Between Exposure to Specific Antibiotic Classes and *Clostridiodes Difficile* Infection (CDI) for Those With Negative Screening Results

	**Original CDI definition**		**Toxin Positive**	
Exposure	Any Exposure	Number of Days	Any Exposure	Number of Days
Aminoglycosides	1.87 (0.71-4.95)	1 (0.84-1.19)	2.1 (0.23-19.5)	0.85 (0.58-1.24)
Amoxicillin/Clavulanate	2.21 (1.13-4.34)	1.14 (1.02-1.27)	2.62 (0.65-10.23)	1.13 (0.91-1.4)
First and second generation Cephalosporins	1.31 (0.44-3.93)	1.01 (0.86-1.19)	1.13 (0.13-10.05)	0.98 (0.75-1.29)
Third and fourth generation Cephalosporins	1.76 (0.97-3.2)	1.08 (0.99-1.18)	1.85 (0.51-6.75)	1.1 (0.94-1.28)
Carbapenem	0.95 (0.37-2.4)	0.93 (0.79-1.09)	--	--
Clindamycin	0.6 (0.08-4.76)	0.89 (0.61-1.31)	--	--
Piperacillin/Tazobactam	2.13 (1.09-4.16)	1.11 (1.01-1.21)	1.03 (0.18-6.04)	1.04 (0.83-1.31)
Other Penicillins	0.79 (0.23-2.73)	0.46 (0.16-1.29)	0.91 (0.1-8.55)	1.08 (0.85-1.38)
Quinolones	0.79 (0.39-1.62)	1.01 (0.89-1.13)	0.63 (0.14-2.96)	1 (0.78-1.29)

© 2025 Gilboa M et al. *JAMA Network Open*.

	Original CDI definition		Toxin Positive	
Exposure	Any Exposure	Number of Days	Any Exposure	Number of Days
Vancomycin IV	1.36 (0.6-3.06)	1.08 (0.92-1.27)	1.23 (0.15-10.22)	1.03 (0.71-1.51)
Other antibiotics	0.84 (0.33-2.12)	0.99 (0.8-1.21)	1.4 (0.3-6.49)	1.17 (0.93-1.47)

CI – confidence interval

Hazard ratios from Cox models adjusted for age, sex, Charlson score, functional status, immunosuppression, and PPI use. CDI was defined per CDC testing criteria. See Methods for full diagnostic definitions.

© 2025 Gilboa M et al. *JAMA Network Open*.

eTable 4. Sensitivity Analysis Accounting for 80% Assay Sensitivity: Adjusted Hazard Ratios for CDI by Antibiotic Exposure Across 10 Iterations

Iteration	Exposure	Crude Hazard Ratio (95% CI)	Adjusted Hazard Ratio (95% CI)
**1**	Any antibiotic treatment (Binary)	1.23(0.69-2.07)	1.2 (0.69-2.1)
	Number of treatment days (Continuous)	1.05 (0.69-2.11)	1.05 (0.99-1.13)
	Number of antibiotic drugs (Continuous)	1.07(0.9-1.28)	1.07(0.91-1.27)
2	Any antibiotic treatment (Binary)	1.19 (0.67-2.12)	1.18 (0.67-2.07)
	Number of treatment days (Continuous)	1.04 (0.97-1.12)	1.05 (0.98-1.12)
	Number of antibiotic drugs (Continuous)	1.05 (0.87-1.25)	1.05 (0.88-1.26)
**3**	Any antibiotic treatment (Binary)	1.11 (0.64-1.94)	1.11 (0.66-1.9)
	Number of treatment days (Continuous)	1.04 (0.97-1.12)	1.04 (0.98-1.12)
	Number of antibiotic drugs (Continuous)	1.05 (0.87-1.26)	1.06 (0.88-1.27)
**4**	Any antibiotic treatment (Binary)	1.15 (0.65-2.06)	1.15 (0.65-2.01)
	Number of treatment days (Continuous)	1.04 (0.97-1.12)	1.05 (0.98-1.12)
	Number of antibiotic drugs (Continuous)	1.05 (0.87-1.26)	1.06 (0.88-1.27)

© 2025 Gilboa M et al. *JAMA Network Open*.

Iteration	Exposure	Crude Hazard Ratio (95% CI)	Adjusted Hazard Ratio (95% CI)
**5**	Any antibiotic treatment (Binary)	1.21 (0.68-2.15)	1.24 (0.7-2.17)
	Number of treatment days (Continuous)	1.04 (0.98-1.12)	1.05 (0.98-1.11)
	Number of antibiotic drugs (Continuous)	1.06 (0.88-1.26)	1.08 (0.9-1.29)
**6**	Any antibiotic treatment (Binary)	1.13 (0.64-2.02)	1.12 (0.64-1.97)
	Number of treatment days (Continuous)	1.04 (0.97-1.12)	1.04 (0.98-1.12)
	Number of antibiotic drugs (Continuous)	1.03 (0.86-1.24)	1.04 (0.87-1.24)
**7**	Any antibiotic treatment (Binary)	1.14 (0.64-2.02)	1.13 (0.64-1.98)
	Number of treatment days (Continuous)	1.04 (0.97-1.11)	1.05 (0.98-1.12)
	Number of antibiotic drugs (Continuous)	1.03 (0.86-1.24)	1.04 (0.87-1.25)
**8**	Any antibiotic treatment (Binary)	1.17 (0.66-2.09)	1.17 (0.66-2.05)
	Number of treatment days (Continuous)	1.04 (0.97-1.12)	1.05 (0.98-1.12)
	Number of antibiotic drugs (Continuous)	1.05 (0.87-1.26)	1.06 (0.89-1.26)
**9**	Any antibiotic treatment (Binary)	1.14 (0.64-2.03)	1.14 (0.65-2)
	Number of treatment days (Continuous)	1.04 (0.97-1.11)	1.04 (0.97-1.11)

© 2025 Gilboa M et al. *JAMA Network Open*.

Iteration	Exposure	Crude Hazard Ratio (95% CI)	Adjusted Hazard Ratio (95% CI)
	Number of antibiotic drugs (Continuous)	1.04 (0.86-1.24)	1.05 (0.87-1.25)
**10**	Any antibiotic treatment (Binary)	1.14 (0.64-2.04)	1.12 (0.64-1.96)
	Number of treatment days (Continuous)	1.04 (0.97-1.11)	1.04 (0.97-1.11)
	Number of antibiotic drugs (Continuous)	1.03 (0.86-1.23)	1.03 (0.86-1.23)

A quantitative bias analysis accounting for the imperfect sensitivity of the screening assay. We assumed 80% sensitivity and perfect specificity. Because we had 1624 positive screens, this would entail 406 false negative results. Thus, in each iteration we randomly sampled 406 hospitalizations and redid the analysis assuming they too were screened positive. Only the analyses with the screening as the exposure or the positive screened as the population were repeated. Overall, 10 analysis iterations were performed.

© 2025 Gilboa M et al. *JAMA Network Open*.

eTable 5. Association Between Exposure to Any Antibiotic and *Clostridioides Difficile* Infection (CDI), Only the First Hospitalization for Each Patient Included

Exposure	Hazard Ratio (95% CI)
**Original CDI Definition**	
**Entire Population**	
Any antibiotic treatment (Binary)	2.11 (1.2-3.7)
Number of treatment days (Continuous)	1.06 (1.01-1.12)
Number of antibiotic drugs (Continuous)	1.22 (1.05-1.41)
**Positive CD Screen**	
Any antibiotic treatment (Binary)	1 (0.52-1.93)
Number of treatment days (Continuous)	0.99 (0.92-1.07)
Number of antibiotic drugs (Continuous)	0.92 (0.75-1.13)
**Negative CD Screen**	
Any antibiotic treatment (Binary)	4.09 (1.27-13.12)
Number of treatment days (Continuous)	1.11 (1.02-1.22)
Number of antibiotic drugs (Continuous)	1.53 (1.23-1.91)

CI – confidence interval

Hazard ratios from Cox models adjusted for age, sex, Charlson score, functional status, immunosuppression, and PPI use. CDI was defined per CDC testing criteria. See Methods for full diagnostic definitions

© 2025 Gilboa M et al. *JAMA Network Open*.

eTable 6. Association Between Exposure to Specific Antibiotic Classes and *Clostridioides Difficile* Infection (CDI), Only the First Hospitalization for Each Patient Included

	**Entire Population**		**Positive CD Screen**		**Negative CD Screen**	
Exposure	Any Exposure	Number of Days	Any Exposure	Number of Days	Any Exposure	Number of Days
Aminoglycosides	1.5 (0.53-4.29)	0.98 (0.78-1.23)	0.7 (0.08-6.17)	0.61 (0.26-1.4)	1.91 (0.61-6.01)	1.01 (0.82-1.24)
Amoxicillin/Clavulanate	1.81 (1.07-3.07)	1.12 (1.03-1.21)	1.06 (0.56-2.03)	1.06 (0.96-1.16)	2.24 (0.94-5.31)	1.1 (0.94-1.29)
First and second generation Cephalosporins	0.95 (0.34-2.7)	1.04 (0.86-1.26)	0.79 (0.19-3.32)	1.04 (0.78-1.38)	1.31 (0.28-6.13)	1.04 (0.82-1.34)
Third and fourth generation Cephalosporins	1.06 (0.65-1.74)	0.97 (0.87-1.08)	0.68 (0.36-1.29)	0.87 (0.74-1.04)	1.87 (0.88-3.97)	1.06 (0.92-1.21)
Carbapenem	1.65 (0.79-3.44)	1.1 (0.97-1.26)	1.22 (0.39-3.84)	1.13 (0.91-1.41)	1.88 (0.7-5.07)	1.06 (0.92-1.23)
Clindamycin	--	--	--	--	--	--
Other Penicillins	0.74 (0.23-2.4)	1.05 (0.87-1.28)	--	--	1.59 (0.44-5.73)	1.13 (0.96-1.33)
Piperacillin/Tazobactam	2.53 (1.52-4.23)	1.14 (1.07-1.22)	1.54 (0.76-3.12)	1.08 (0.97-1.2)	3.25 (1.47-7.15)	1.15 (1.04-1.27)
Quinolones	1.04 (0.59-1.84)	1.03 (0.93-1.14)	1.15 (0.55-2.42)	1.04 (0.91-1.19)	0.98 (0.41-2.39)	1.05 (0.91-1.22)
Vancomycin IV	0.84 (0.4-1.79)	1 (0.83-1.19)	0.62 (0.2-1.89)	1.01 (0.77-1.32)	1.06 (0.38-2.91)	0.99 (0.82-1.19)

© 2025 Gilboa M et al. *JAMA Network Open*.

	Entire Population		Positive CD Screen		Negative CD Screen	
Exposure	Any Exposure	Number of Days	Any Exposure	Number of Days	Any Exposure	Number of Days
Other antibiotics	0.84 (0.38-1.84)	0.84 (0.67-1.06)	0.9 (0.32-2.54)	0.71 (0.48-1.06)	0.9 (0.27-2.93)	0.95 (0.75-1.2)

Hazard ratios from Cox models adjusted for age, sex, Charlson score, functional status, immunosuppression, and PPI use. “Original” CDI definition: unformed stool with positive PCR and EIA (GDH or toxin). “Specific” definition: positive PCR and EIA for toxin. CDI testing followed CDC guidelines (≥3 unformed stools/24h, no laxatives). “Other antibiotics” include tetracyclines, TMP-SMX, macrolides, and chloramphenicol. “--” indicates insufficient data to estimate effect.

© 2025 Gilboa M et al. *JAMA Network Open*.

eFigure 1. Antibiotic Exposure Histograms

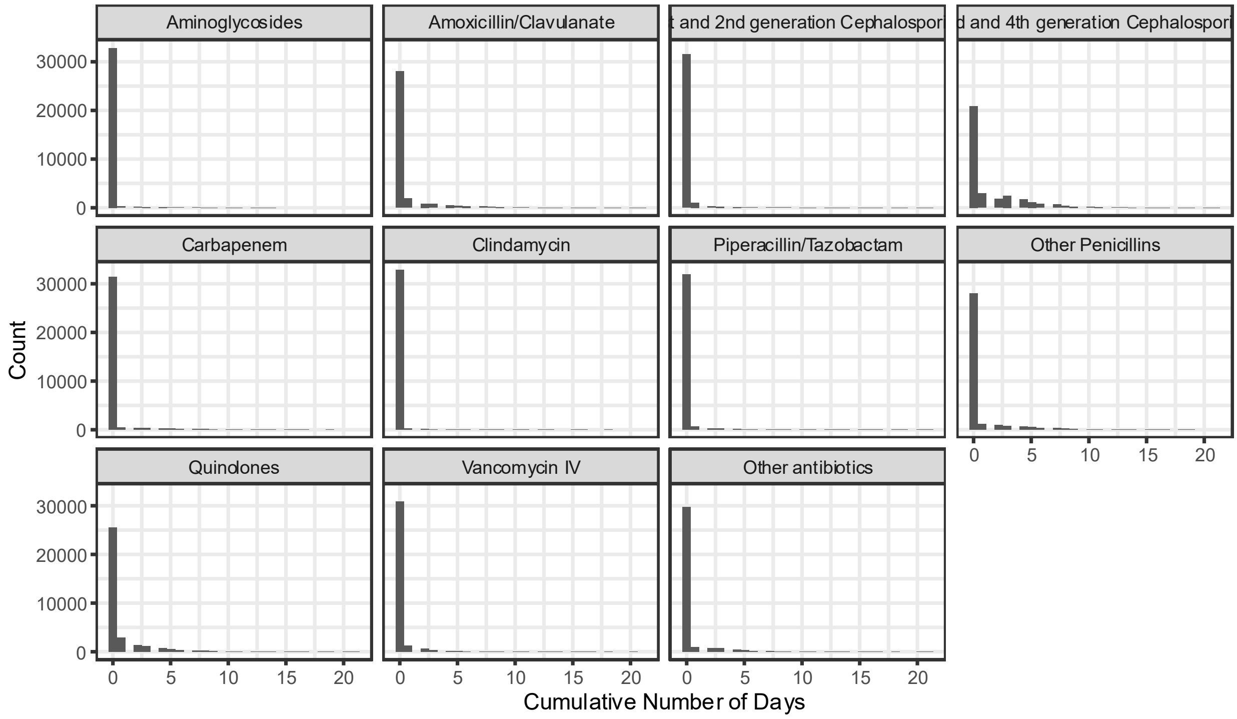

eFigure 1. *Distribution of antibiotic exposure days by antibiotic class.*

This figure presents histograms showing the distribution of the number of days patients were exposed to each antibiotic class during hospitalization. Each subplot represents a different antibiotic class included in the analysis. The x-axis indicates the number of exposure days, and the y-axis indicates the number of hospitalizations. The figure illustrates variability in exposure duration across antibiotic types, with most exposures concentrated within the first few days.

© 2025 Gilboa M et al. *JAMA Network Open*.

eFigure 2. Survival Curves

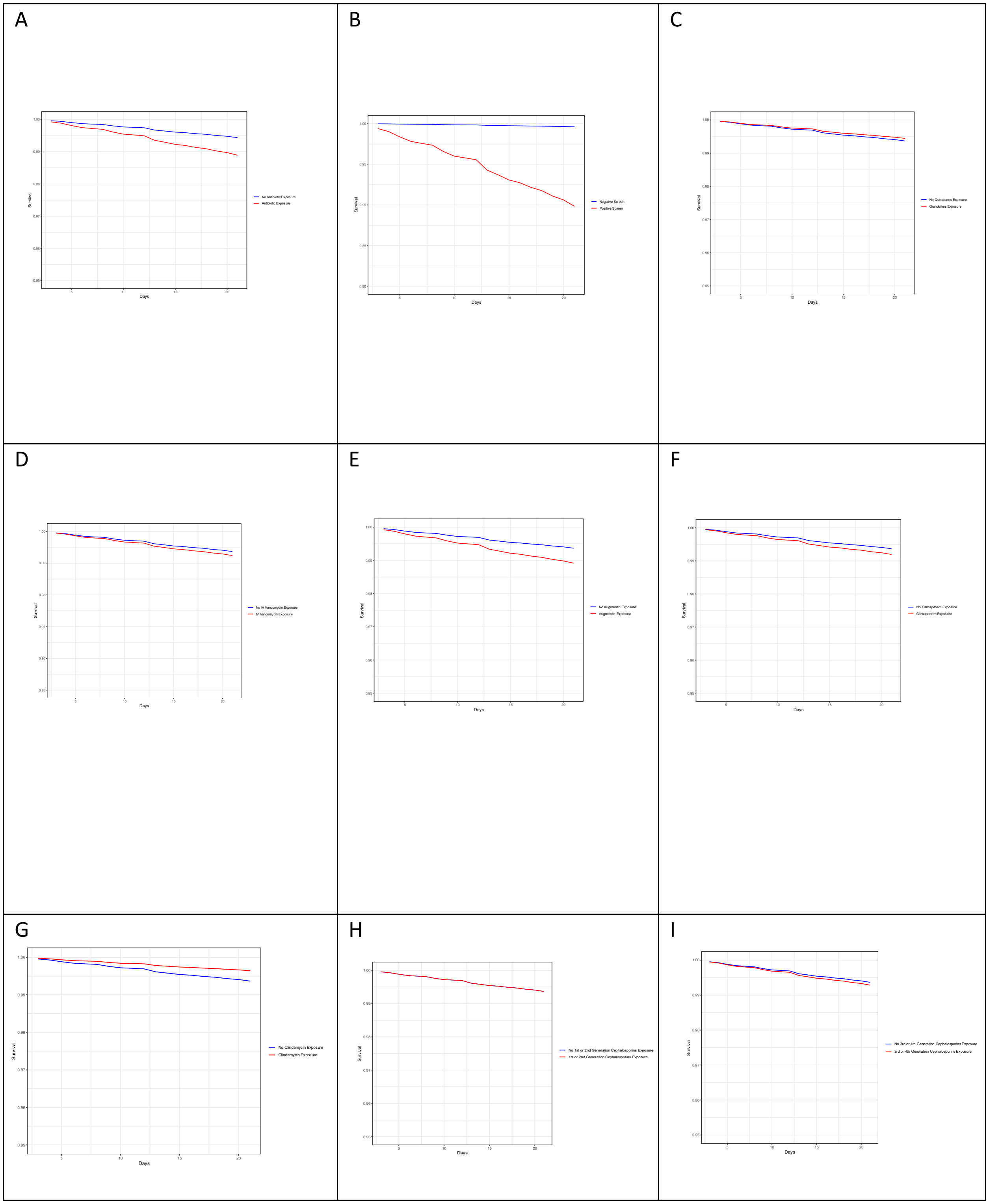

© 2025 Gilboa M et al. *JAMA Network Open*.

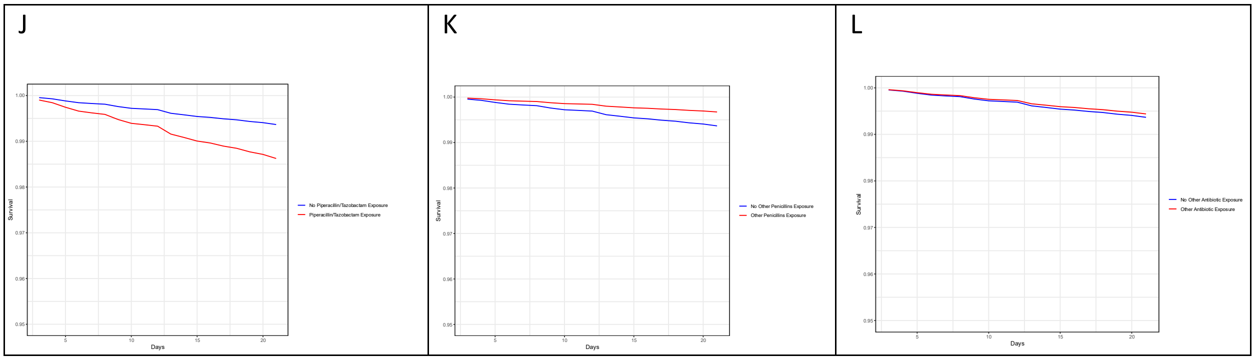

eFigure 2- survival curves comparing patients exposed to antibiotics to patients not exposed. The curves are based on the adjusted model. Representative covariate values were selected, based on the median of the sample (79-year-old, male, Charlson's score 6, bad functional state, not immunosuppressed, not taking PPIs). In each plot, exposure begins at day 3 and lasts until day 21).

Survival curves show time to CDI stratified by 12 exposures:
A – Any antibiotic use,
B – Positive vs. negative C. difficile screen at admission,
C – Quinolones,
D – IV vancomycin,
E – Amoxicillin–clavulanate,
F – Carbapenems,
G – Clindamycin,
H – 1st/2nd generation cephalosporins,
I – 3rd/4th generation cephalosporins.
J- Piperacillin-Tazobactam
K- Other penicillin
L- Other antibiotics

© 2025 Gilboa M et al. *JAMA Network Open*.
