# Supplementary material for: Antibiotic Use and the Risk of Hospital-Onset Clostridioides Difficile Infection
Source: JAMA Netw Open. 2025 Aug 8;8(8):e2525252. doi: 10.1001/jamanetworkopen.2025.25252 (PMC12334957; doi:10.1001/jamanetworkopen.2025.25252)
Supplement: Supplement 2. — Data Sharing Statement [file jamanetwopen-e2525252-s002.pdf]

Data Sharing Statement

Gilboa. Antibiotic Use and the Risk of Hospital-Onset Clostridioides Difficile Infection. *JAMA Netw Open*. Published August 08, 2025. doi:10.1001/jamanetworkopen.2025.25252

Data
Data available: Yes
Data types: Deidentified participant data
How to access data: data can be provided by the corresponding author upon reasonable request
When available: With publication

Supporting Documents
Document types: None

Additional Information
Who can access the data: data can be provided by the corresponding author upon reasonable request
Types of analyses: data can be provided by the corresponding author upon reasonable request
Mechanisms of data availability: data can be provided by the corresponding author upon reasonable request
